# A Comparative Assessment of Pain Caused by the Placement of Banded Orthodontic Appliances with and without Low-Level Laser Therapy: A Randomized Controlled Prospective Study

**DOI:** 10.3390/dj8010024

**Published:** 2020-03-04

**Authors:** Carmelo Nicotra, Alessandro Polizzi, Graziano Zappalà, Alessandro Leonida, Francesco Indelicato, Gianluigi Caccianiga

**Affiliations:** 1 Department of General Surgery and Surgical-Medical Specialties, University of Catania, 95124 Catania, Italy; gnicotra@unict.it (C.N.); grazianozap@live.it (G.Z.); indelicato@policlinico.unict.it (F.I.); 2 School of Medicine and Surgery, University of Milano-Bicocca, 20900 Monza, Italy; leo.doc73@libero.it (A.L.); gianluigi.caccianiga@unimib.it (G.C.)

**Keywords:** low-level laser therapy, orthodontic tooth movement, orthodontics, dental materials, laser, dental pain

## Abstract

Patients still refuse or discontinue orthodontic treatment due to related pain and discomfort. In this study, we investigate if low-level laser therapy (LLLT) can reduce pain caused by orthodontic bands. Sixty subjects who needed bands placed on the upper permanent first molars were assigned randomly to the LLLT group, placebo, and control groups. Inclusion criteria were: age range 10–14 years, fully erupted upper first molars in healthy condition, presence of tight mesial proximal contact. Exclusion criteria were: systemic or metabolic diseases, chronic pain or neurological or psychiatric disorders, use of pharmacological agents interfering with pain perception, previous orthodontic treatment or the simultaneous presence of other devices in the patient’s mouth. The assessment of pain was performed by using a numeric rating scale (NRS) considering different time intervals, i.e., immediately after bands placement, 6 h, 24 h, and from day 2 to day 5. Differences in the maximum pain and in pain experienced at each time-point, among the three groups, was assessed by using the Kruskal–Wallis H. The final sample included 56 patients, 29 males, and 27 females, with a mean age of 12.03 ± 1.3 years. Patients were randomly allocated into three groups (tested, control, and placebo group) with each group consisting respectively of 19, 20, and 17 individuals. Subjects in the LLLT experienced less pain at each time interval as well as the maximum pain score being lower in the LLLT compared to control and placebo groups. These findings were all statistically significant (*p* < 0.05). LLLT can alleviate the intensity of pain after the placement of orthodontic bands.

## 1. Introduction

Although the demand for improving smile aesthetics and occlusal functionality has increased over the last decades, a large percentage of patients, from children to adults, are still concerned about pain and discomfort related to orthodontic treatment [1,2]. This sometimes leads patients to refuse, delay or discontinue the orthodontic treatment [3,4,5].

The pain mechanism in orthodontic treatment is a result of compression forces leading to ischemia, inflammation, and edema in the periodontal tissues [5,6,7]. To obtain pain relief pharmacological agents (nonsteroidal anti-inflammatory drugs and topical anesthetic formulations) have been generally recommended by orthodontists [7,8,9]. However, several nonpharmacological methods for pain relief have also been proposed, such as vibratory stimuli [10], transcutaneous nerve stimulation [11], and, over the years, low-level laser therapy (LLLT) [2,6].

Low-level laser therapy is a non-invasive, side-effect-free therapy that uses a laser light within the red to the near-infrared range (wavelengths from 632 nm to 1064 nm) to provoke a biological reaction [12,13,14]. It has been reported that LLLT can induce significant biological alterations: LLLT seems to increase bone cell (osteoblastic and osteoclastic) and vascular activity and to stimulate collagen production [8,14,15]. This results in an overall increase in bone metabolism. LLLT also has analgesic and anti-inflammatory effects expressed as an increase in the local blood flow (vasodilatation) and inhibition of the mechanism (COX2-2: cycloxygenase-2) that leads to the release of prostaglandin E2 from arachidonic acid. Moreover, the use of the laser causes the release of beta- endorphin, which induces an effective analgesic reaction [8,14,16,17].

In particular, in the orthodontic field, it was shown that LLLT effectively increases the rate of orthodontic movement during the alignment stage and canine retraction as well as reducing the pain experienced in the days after the engagement of active orthodontic alignment archwire [18,19,20]. However, low quality of evidence has supported LLLT to accelerate orthodontic tooth movement and modulate acute orthodontic pain. In this respect, the need for high-quality research, with consistency in study design, has been claimed to be needed to determine whether LLLT can enhance the patients’ experience of the orthodontic treatment [18,21].

Although the use of orthodontic bands has been significantly reduced compared to the past, still today, many orthodontic/orthopedic devices need bands on the “anchored” teeth to enable stable retention in the mouth. However, the placement of molar bands is painful and not well tolerated especially by young patients [1,4,21]. Thus, the present randomized controlled study aimed to assess if LLLT could reduce the pain experienced by patients after the insertion of orthodontic bands (also known as molar bands). The null hypothesis was the absence of difference in pain experienced after placement of orthodontic bands between subjects who underwent LLLT and controls.

## 2. Materials and Methods

### 2.1. Study Design

Sixty subjects were recruited from a larger sample (106) of patients seeking orthodontic treatment. A preliminary power analysis showed that 20 participants (10 for each group) were required to detect a difference of 3 points in the pain experienced after placement of separator elastics between LLLT and control groups. Nevertheless, to increase the robustness of the data, we included 20 subjects in each group (tested, control, and placebo). The study was approved by the local IRB of the University of Catania (18/18—23 March 2018). The study was registered on clinicaltrials.gov (NCT03873879). All patients who voluntarily agreed to participate also signed appropriate informed consent.

Patients were enrolled according to the following inclusion criteria: age between 10 and 14 years, completely erupted and healthy maxillary permanent first molars (absence of caries and periodontal disease), presence of tight mesial proximal contacts of maxillary permanent first molars, orthodontic treatment plan involving the use of maxillary device anchored on both right and left molars (maxillary expander, transpalatal arch). Exclusion criteria were: Systemic or metabolic diseases that would contraindicate the use of LLLT, chronic pain or neurological or psychiatric disorders, use of pharmacological agents interfering with pain perception, and the use of analgesic during the time test, previous orthodontic treatment or the simultaneous presence of other devices in the patient’s mouth.

A randomized balanced block protocol using sex as a stratification factor was performed to randomly allocate subjects to receive LLLT (20 subjects) and to be included in the control group (no administration of LLLT, 20 subjects) and placebo group (simulated administration of LLLT, 20 subjects). In the placebo group, the device was turned off unbeknown to the blinded subjects included in this group. The SPSS Statistics software (IBM Corporation, Armonk, NY, USA) was used to generate an allocation sequence.

Administration of LLLT (tested group, 30 subjects), with a fixed appliance and simulated administration of LLLT (placebo group, 30 subjects) and with fixed appliance only (control group, 30 subjects).

### 2.2. Intervention

The orthodontic device, i.e., maxillary expander or transpalatal arch, was inserted firstly pressing the bands against the interproximal contacts with fingers, then while holding the bands with the Weingart plier, significant pressure was applied in order to separate the interproximal contacts until the bands slid down until 1 mm below the marginal crest [22]. The device was cemented by using fluoride cement (Ketac Cem, 3M, Saint Paul, MN, USA). The appliance was kept passive, i.e., without applying active forces, up to the end of the time-line schedule, in order to avoid confounding factors that could have altered the perception of pain.

**Figure 1:**
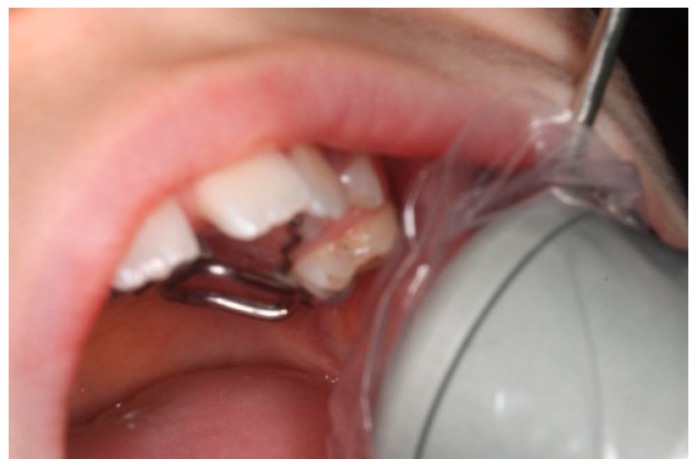
Low-level laser therapy (LLLT) administration. The optical fiber tip was moved from vestibular toward the palatal side of the upper first molar for 10 s (each session).

On the same day, after placement of the orthodontic device, LLLT (AlGaAs diode laser emitting infrared radiation at 980 nm) was administered in the office. The size of the spot tip was (1 cm^2^) and laser irradiation was performed in continuous wave mode (1W output power, 1J/cm^2^ energy density per point) by positioning the optical fiber tip over the first molar on both sides (anchoring teeth) with a single spot application moving the tip from vestibular toward the palatal side for 10 s. The procedure was repeated 3 times at an interval of 10 s. Thus, each molar received a total fluence of 30J/cm^2^. (Figure 1). The procedure of data collection was entrusted to another operator (G.C.).

### 2.3. Assessment of Pain

All patients were instructed to report perceived pain by using NRS (Numerical Rating Scale), from 0 = (no pain) to 10 (maximum pain) referring to specific time intervals, i.e., immediately after bands cementation, 6 h, 24 h, and from day 2 to day 5 [23]. Except for the first pain reported, which was recorded in-office, all patients were forewarned at each time by sending them a google form link via What’UP where they could easily visualize the NRS scale and report pain experienced adequately. Subjects were instructed to complete the online form in the morning after breakfast, in order to avoid other mood bias throughout the day. Moreover, subjects were instructed to avoid analgesics and to report the consumption of drugs in the online form answering to a specific query. The data collection process was entrusted to another operator (A.L.).

### 2.4. Statistical Analysis

The Shapiro–Wilk test [24] was used to assess the normality distribution of data. Since data were not normally distributed non-parametric tests were used to perform inferential statistics. In particular, the Kruskal–Wallis H test was used to evaluate if subjects in tested, control and placebo groups experienced different levels of pain at each time interval and to compare the maximum pain score reported among the three groups.

## 3. Results

**Figure 2:**
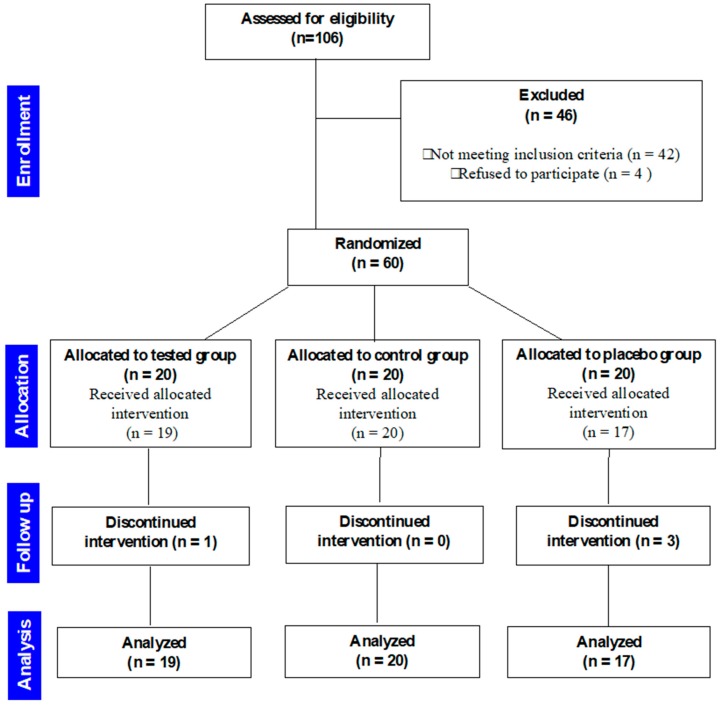
CONSORT flowchart.

Four subjects were excluded from the final study sample: three participants in the PG used anti- inflammatory drugs to manage pain due to excessive pain. One patient in the TG group discontinued reporting pain in the appropriate Google Form. As a result, the final sample consisted of 56 patients, 29 male, and 27 female, mean age 12.03 ± 1.30. Demographic and descriptive statistics of the study sample are reported in Table 1 and Figure 2, which shows the study sample flowchart (CONSORT).

**Figure 3:**
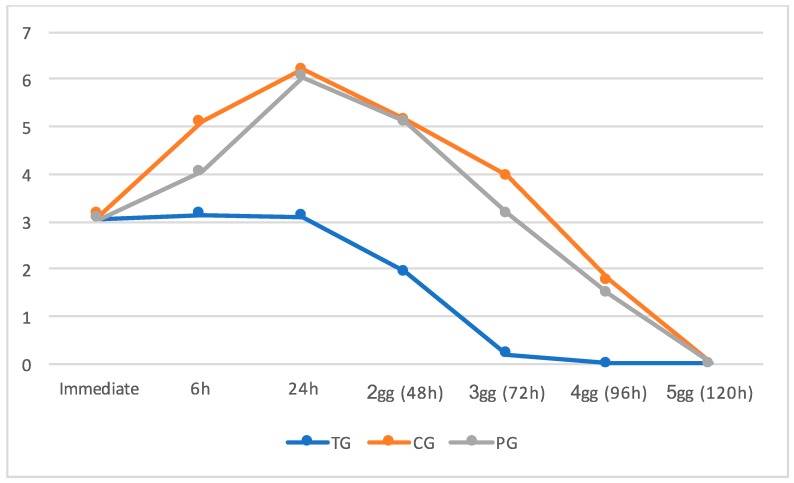
Graphic representation of the level of pain experienced at each time interval in both treatment group (TG), control group (CG), and placebo group (PG).

According to our findings, in the TG, the pain experienced by the patients was maximum but sustained from T0 (immediate) to T2 (24 h) after the insertion of the orthodontic bands, with a moderate-low score of 3, and then it rapidly decreased to a low pain score of 2 at 48 h (T3) and 0 on the following days (from T4 to T6). In the PG, maximum pain score was reported 24 h (T2) after the insertion of the molar bands with a median score of 6, and then pain gradually decreased to a median score of 5 on day 2 (T3), 3 on day 3(T4), 1 on day 4 (T5), and 0 after 120 h (T6) In the CG, pain perceived was maximum at 24 h (T2) (immediate) after the insertion of the molar bands with a median score of 6, and then, pain slowly decreased to a median score of 5 on day 2 (T3), 4 on day 3 (T4), 2 on day 4 (T5), and finally 0 after 120 h (T6) (Table 2). Figure 3 shows the trend of pain through the investigated time period in each group.

The pain score reported significantly differs among the three groups at time intervals except for T0 and T6 timeline where no significant difference in pain perception was reported. In particular, pain experienced in the TG was significantly lower compared to both control and placebo groups, while control and placebo groups showed no differences, according to Dunn’s multiple comparison test (Table 2, Figure 3).

## 4. Discussion

This study comparatively assessed the pain experienced after the placement of orthodontic bands in patients receiving LLLT and control group. Our findings are related to a sample of Caucasian children/young adolescents living in good social and environmental conditions, thus they cannot be generalized to a population of subjects reporting different socio-economics conditions.

The results of the present study showed that pain started immediately after the insertion of orthodontic bands with subjects in all groups reporting a similar mild value of pain (median value 3). The pain started as soon as bands were placed because (1) the immediate and forced separation of inter-proximal contact that induced stretching of periodontal fibers [25] and (2) pressure on the gingiva was unavoidable although bands were drifted within the free gingival sulcus.

However, while subjects in TG showed a progressive and constant relief from pain, subjects in both control and placebo groups reported higher values of pain in the following timelines, in particular reaching the highest median value at 24 h (median value 6). After this timeline, a sharp decrease of pain was recorded at 96 h, reaching baseline levels at 120 h.

From a clinical perspective, a reduction of two points in the scale of pain intensity is the benchmark of a significant change, according to the studies of Farrar [25,26]. In this regard, the differences between tested and control group in both maximum pain score and in the pain recorded at specific time-lines (6 h, 24 h, day 2, 3 and 4) were equal or higher than two points on the NRS scale (Table 2), corroborating the clinical relevance of our findings. In this respect, LLLT can be administered to patients before placing an orthodontic band since this can enhance their experience of treatment. Our findings corroborate previous research on the efficacy of LLLT in controlling pain during orthodontic treatment. In particular, Lo Giudice et al. [2] demonstrated that LLLT can be used to reduce the intensity and duration of pain the engagement/activation of orthodontic alignment arch-wire. Moreover, other studies on some oral procedures, have suggested that LLLT can alleviate the intensity of pain during the different stages of orthodontic treatment, including the space closure stage [27,28,29,30,31,32].

Considering the differences between the experimental and placebo groups in the present study, our findings confirm that LLLT does not induce psychological effects that may reduce the perception of pain, as previously demonstrated [33,34,35].

It has been reported that a high dosage should be avoided in inflammatory pain because it reduces the anti-inflammatory and analgesic effects, while increasing tissue heat [33]. In particular, a dosage 20J/cm^2^ per area and 5J/cm^2^ per point [36,37,38] was suggested in some studies on the treatment of oral diseases, to effectively reduce pain after placement of elastic separators. Also, Almallah et al. [38] used LLLT with a dosage of 112J/cm^2^ and found a significant reduction of pain after the insertion of elastomeric separators. In this study, we used a fluency of 30 J cm^2^ which is slightly over this range. In this respect, it is still not fully understood how LLLT [39,40,41,42,43,44,45], and different range of administration of LLLT, can alter and mitigate nociception [46,47,48,49,50,51,52,53,54]. However, the present study confirms that LLLT effectively mitigates orthodontic pain [55,56,57,58,59,60], this corroborates previous studies showing that it moderates the levels of prostaglandin E2 and stimulates the production of beta-endorphin and several other mediators [12,14,40,49,61,62,63,64,65,66].

Despite the placement of elastics separators being conventionally used to slowly open the interproximal contacts in order to reduce discomfort during the placement of orthodontic bands, we decided to investigate directly the pain experienced without open space preparation. Considering the low level of pain reported by subjects who underwent LLLT in our study, clinicians may consider they do not require a specific appointment for place elastic separators, thus reducing chairside time.

## 5. Limitations

The main limitation of the study is related to the absence of strict control during the compilation of the questionnaire, thus our findings rely on the authenticity of patients’ behavior. However, this is an intrinsic limitation of on-line questionnaire-based studies and any efforts have been computed to standardize the data acquisition.

Furthermore, future studies are required in order to assess the most effective LLLT protocol for the management of orthodontic pain in both youngsters and adults.

## 6. Conclusions

The results of the present study evidenced that LLLT reduces the intensity of pain reported by patients after the placement of orthodontic bands. In this respect, clinicians may consider using this protocol to enhance the patients’ experience at a chairside time.

## Figures and Tables

**Table 1:** Demography, clinical characteristics and descriptive statistics of the study sample. TG = Tested group, CG = Control Group, PG = Placebo Group. * *p*-value set as ≤0.05. Assessed by paired *t*- test or chi-square test. NS = not significant.

Sample Characteristics	Total Sample (n = 56)	TG (n = 19)	CG (n = 17)	PG (n = 20)	Significance *
**Gender: male/female**	29/27	9/10	10/7	8/12	NS
**Age, year: mean (SD)**	12.03 (1.30)	12.05(1.31)	12.41 (1.32)	11.7 (1.26)	NS

**Table 2:** The maximum pain score and pain experienced at each schedule, assessed via the Numeric Rating Scale (NRS). TG = Tested group, CG = Control Group, PG = Placebo Group. MPS = Maximum Pain Score. * *p*-value set as ≤0.05. Assessed by Kruskal–Wallis H Test.

Time Schedule	TG (n = 27) Median (Min-Max)	CG (n = 29) Median (Min-Max)	PG (n = 28) Median (Min-Max)	Significance *
MPS	4 (2–4), 95% CI: 3.05–3.79	6 (5–7), 95% CI: 5.72–6.52	6 (5–7), 95% CI: 5.87–6.53	<0.001
Immediate	3 (2–4), 95% CI: 2.75–3.35	3 (2–4), 95% CI: 2.67–3.44	3 (2–4), 95% CI: 2.80–3.50	NS
6 h	3 (2–4), 95% CI: 2.83–3.49	4 (3–5), 95% CI: 3.67–4.44	5 (4–6), 95% CI: 4.76–5.44	<0.001
24 h	3 (2–4), 95% CI: 2.72–3.50	6 (5–7), 95% CI: 5.67–6.44	6 (5–7), 95% CI: 5.87–6.53	<0.001
48 h	2 (1–3), 95% CI: 1.65–2.25	5 (4–6), 95% CI: 4.76–5.48	5 (3–7), 95% CI: 4.64–5.66	<0.001
72 h	0 (0–1), 95% CI: 0.01–0.41	3 (2–5), 95% CI: 2.72–3.63	4 (1–5), 95% CI: 3.44–4.46	<0.001
96 h	0 (0–0), 95% CI: 0.00–0.00	1 (0–3), 95% CI: 1.10–1.84	2 (0–3), 95% CI: 1.35–2.15	<0.001
120 h	0 (0–0), 95% CI: 0.00–0.00	0 (0–0), 95% CI: 0.00–0.00	0 (0–0), 95% CI: 0.00–0.00	Not Significant
